# Betaglycan Gene (*TGFBR3*) Polymorphism Is Associated with Increased Risk of Endometrial Cancer

**DOI:** 10.3390/jcm9103082

**Published:** 2020-09-24

**Authors:** Piotr K. Zakrzewski, Ewa Forma, Adam I. Cygankiewicz, Magdalena Bryś, Katarzyna Wójcik-Krowiranda, Andrzej Bieńkiewicz, Andrzej Semczuk, Wanda M. Krajewska

**Affiliations:** 1 Department of Cytobiochemistry, Faculty of Biology and Environmental Protection, University of Lodz, Pomorska 141/143, 90-236 Lodz, Poland; ewa.forma@biol.uni.lodz.pl (E.F.); adam.cygankiewicz@biol.uni.lodz.pl (A.I.C.); magdalena.brys@biol.uni.lodz.pl (M.B.); wanda.krajewska@biol.uni.lodz.pl (W.M.K.); 2 Department of Gynecological Oncology, Medical University of Lodz, Pabianicka 62, 93-513 Lodz, Poland; kkrowiranda@o2.pl (K.W.-K.); abienkiewicz@wp.pl (A.B.); 3 IInd Department of Gynecology, Medical University of Lublin, Jaczewskiego 8, 20-090 Lublin, Poland; andrzejsemczuk@umlub.pl

**Keywords:** SNP, TGFβ, *TGFBR*3, betaglycan, endometrial cancer

## Abstract

We investigated single nucleotide polymorphism (SNP) of the betaglycan gene (*TGFBR3*) encoding the TGFβ co-receptor in endometrial cancer (EC) and its association with betaglycan expression. The study group included 153 women diagnosed with EC and 248 cancer-free controls. SNP genotyping and gene expression were analyzed using TaqMan probes. Three out of the eight SNPs tested, i.e., *rs12566180* (CT; OR = 2.22; 95% CI = 1.15–4.30; *p* = 0.0177), *rs6680463* (GC; OR = 2.34; 95% CI = 1.20–4.53; *p* = 0.0120) and *rs2296621* (TT; OR = 6.40; 95% CI = 1.18–34.84; *p* = 0.0317) were found to be significantly associated with increased risk of EC (adjusted to age, body mass index, menarche and parity). Among the analyzed SNPs, only *rs2296621* demonstrated the impact on the increased cancer aggressiveness evaluated by the WHO grading system (G3 vs. G1/2, GT—OR = 4.04; 95% CI = 1.56–10.51; *p* = 0.0026; T—OR = 2.38; 95% CI = 1.16–4.85; *p* = 0.0151). Linkage disequilibrium (LD) analysis revealed high LD (r2 ≥ 0.8) in two haploblocks, constructed by *rs2770186*/*rs12141128* and *rs12566180*/*rs6680463*, respectively. In the case of C/C haplotype (OR = 4.82; 95% CI = 1.54–15.07; *p* = 0.0116—Bonferroni corrected) and T/G haplotype (OR = 3.25; 95% CI = 1.29–8.15; *p* = 0.0328—Bonferroni corrected) in haploblock *rs12566180*/*rs6680463*, significantly higher frequency was observed in patients with EC as compared to the control group. The genotype-phenotype studies showed that SNPs of the *TGFBR*3 gene associated with an increased risk of EC, i.e., *rs12566180* and *rs2296621* may affect betaglycan expression at the transcriptomic level (*rs12566180*—CC vs. TT, *p* < 0.01; *rs2296621*—GG vs. TT, *p* < 0.001, GT vs. TT, *p* < 0.05). Functional consequences of evaluated *TGFBR*3 gene SNPs were supported by RegulomeDB search. In conclusion, polymorphism of the *TGFBR*3 gene may be associated with an increased EC occurrence, as well as may be the molecular mechanism responsible for observed betaglycan down-regulation in EC patients.

## 1. Introduction

Endometrial cancer (EC) is one of the leading female cancer-related causes of death with around 382,069 new cases and 89,929 deaths worldwide each year. Significantly higher incidence rate is observed in developed countries in contrast to less-developed ones with the world morbidity around 8.4/100,000 of female population [1]. In Poland, its incidence rate takes the third position among cancers and occurs predominantly in women in their menopausal and post-menopausal age (30.2/100,000). However, EC occurrence in Poland has risen rapidly in the last three decades, although the mortality trend is stable, as it is observed in global population [2]. In the future, the incidence of endometrial cancer is expected to increase due to the gradual aging of the population.

According to the clinico-pathological features and different pathogenesis, endometrial cancer is commonly classified into type I—endometrioid and type II—non-endometrioid. Type I is the most diagnosed type of endometrial cancer (75–90%) and develops from glandular cells in the endometrium lining. Endometrioid tumors are represented predominantly by endometrial adenocarcinomas, which are estrogen-dependent and tend to be low grade with favorable prognosis. Non-endometrioid cancers typically include papillary serous or clear cell carcinomas, in general, histological subtypes characterized by more aggressive phenotypes with poor outcome [3,4]. Endometrial cancer is mostly diagnosed in the early stages (FIGO I and II) as it is observed in 75% of patients. In this stage, 5-year overall survival is 74–91%, whereas for more advanced stages, 5-year overall survival rates are 57–66% and 20–26%, for FIGO III and FIGO IV, respectively [5]. Molecular classification distinguished based on a large scale, comprehensive genetic analysis of EC according to The Cancer Genome Atlas includes four subgroups, i.e., DNA polymerase epsilon ultramutated (*POLE*), microsatellite instability hypermutated (MSI), copy-number low and copy-number high subgroup. Each of categories is characterized by distinct clinical, pathological and molecular alterations. The POLE subgroup displays polymerase epsilon mutations in exonuclease domain, which results in a remarkable high mutation rate (232 × 10^−6^ mutations per Mb). The MSI subgroup is related to deficiencies in a DNA mismatch repair system leading to common mutations of *ARID*5*B*, *PTEN*, *PIK*3*CA* and *PIK*3*R*1 genes. The copy-number low subgroup is described also as microsatellite stable and corresponds to more than half of low-grade endometrioid tumors, whereas copy-number high subgroup reflects to serous histopathology [6,7,8]. Moreover, up to 5% of ECs are described as familial ones, due to the loss-of-function or expression alterations of DNA mismatch repair genes, i.e., (*MLH*1, *MSH*2, *MSH*6 or *PMS*2). The most frequent form of inherited EC is associated with Lynch syndrome, which increases the risk of developing EC to 25–60% [9,10].

According to the molecular findings, impaired TGFβ signaling has been reported in ECs [11,12,13]. The canonical signal, mediated via TGFβ factors, occurs through TGFβ membrane receptors type I (TGFβRI) and type II (TGFβRII), which possess serine/threonine kinase activity. Among huge number of TGFβ factors, three classical TGFβ isoforms, i.e., TGFβ1, TGFβ2 and TGFβ3, were identified. Dimeric TGFβ factors bind to the TGFβRII receptor, which in turn activates TGFβRI receptor. The activated TGFβRII/TGFβRI complex trans-phosphorylates cytoplasmic effectors, i.e., Smad2/3 proteins, forming a heterocomplex with the Smad4 protein, are translocated to the nucleus, where together with other transcription factors, regulate gene expression [14]. Appropriate signaling in TGFβ pathway requires the presence of co-receptors termed TGFβ receptors type III (TGFβRIII). TGFβ co-receptors are deprived of any known enzymatic activity; however, they are anchored in the cell membrane, and they are responsible for TGFβ ligand presentation to their canonical TGFβ receptors. The plethora of TGFβ ligands and receptors results in the regulation of many cellular processes, such as growth and proliferation, survival, apoptosis, cells adhesion, remodeling of extracellular matrix, angiogenesis and embryonic development [15].

The first identified TGFβ co-receptor was betaglycan [16]. Betaglycan gene (*TGFBR*3), located on chromosome 1, encodes a transmembrane proteoglycan. Literature data and our previous studies indicate the contribution of betaglycan loss to the development and progression of cancers originated from different tissue types, i.e., breast, endometrium, ovary, prostate, lung, bladder, liver, pancreas, kidney, and neuroblastoma [17,18,19,20,21,22,23,24,25,26,27,28]. Down-regulation of betaglycan expression seems to be engaged in the impaired TGFβ signaling initiated by the TGFβ2 isoform to which it displays the highest affinity.

Until now, there is no efficient and rapid molecular method suitable for neither early diagnosis nor prediction of EC risk. This became a basis for betaglycan (*TGFBR*3) gene single nucleotide polymorphism (SNP) investigations. In the current study, we examined eight SNPs within the *TGFBR*3 gene and their association with primary EC and their clinico-pathological variables, as well as potential impact on betaglycan expression.

## 2. Materials and Methods

### 2.1. Study Population

In the study, we enrolled only Caucasian women born and living in Poland. The case-control study involved 153 women who underwent surgery of EC and 248 healthy individuals which served as cancer-free controls. Biological material (cancer group—endometrial tissue samples and peripheral blood; cancer-free control—peripheral blood) was collected in the II^nd^ Department of Gynecology, Lublin Medical University, Lublin, Poland and in the Department of Gynecological Oncology, Medical University of Lodz, Lodz, Poland, between 2012–2017. Inclusion criteria for case group, included women with diagnosed primary endometrial adenocarcinomas, who had not received neither hormonal therapy, radiation therapy nor chemotherapy prior surgery; whereas control group was recruited from non-related women during periodic health check-ups, who have never been diagnosed with endometrial cancer or other tumors.

Cancer tissue specimens, after the surgery, were divided into two portions; one was fixed in buffered formalin (pH 7.4) for routine histological assessment while the other was immediately placed at −70 °C. Clinical stage was assigned based on surgico-pathological findings according to the revised FIGO staging, while WHO classification was applied to determine the histological type and grade. Table 1 presents socio-demographic and clinical characteristics of the patients and examined samples. All studied cancer samples were classified as endometrioid cancers (type I)—endometrial adenocarcinomas.

### 2.2. Ethical Approval

The study was conducted in accordance with the ethical principles of the 1975 Helsinki Declaration and its later amendments. The local Independent Committees of Bioethics of Lublin Medical University, Medical University of Lodz and University of Lodz approved the tissues collections and study protocols. All methods in the study were performed in accordance with above-mentioned bioethical permissions. All participating subject gave written, informed consent prior to enrolment.

### 2.3. Lifestyle Risk Factors

Study participants were interviewed during the examination about socio-demographic, health related information and reproductive history (parity and menarche). Body mass index (BMI) was calculated as current weight in kilograms divided by square of height expressed in meters. Any missing survey data were subsequently completed using patient’s query.

### 2.4. Genomic DNA Isolation

Genomic DNA was extracted from peripheral blood collected in the presence of anti-coagulant (EDTA) using PureLink Genomic DNA Mini Kit (Thermo Fisher Scientific, Waltham, MA, USA) and stored at −70 °C. The quality and quantity of DNA was estimated spectrophotometrically with BioPhotometer plus (Eppendorf, Hamburg, Germany). DNA samples were characterized with A260 nm/A280 nm ratio, which was in the range of 1.8–2.0.

### 2.5. SNP Selection and Genotyping

Eight SNPs in the *TGFBR*3 gene were selected according to NCBI SNPs database: *rs883873*, *rs2770186*, *rs12141128*, *rs12566180*, *rs6680463*, *rs1805110*, *rs1805113*, *rs2296621*. All SNPs were supposed to have minor allele frequency (MAF) ≥5% and localization assigned as 5′ regulatory region, intron or exon of the *TGFBR*3 gene, which is located on chromosome 1. Characteristics of studied SNPs are presented in Table 2.

Real-Time PCR method with TaqMan Genotyping Assays (Thermo Fisher Scientific, Waltham, MA, USA) was applied for SNPs genotyping. The characteristics and sequences of used TaqMan probes are shown in Supplementary Table S1. PCR amplifications were conducted in a total volume of 10 µL and consisted of 5 µL (2×) of TaqMan Genotyping Master Mix buffer (Thermo Fisher Scientific, Waltham, MA, USA), 0.25 µL (40×) TaqMan Genotyping Assay (Thermo Fisher Scientific, Waltham, MA, USA) and 10 ng of template DNA. Thermal conditions were as follows: initial denaturation at 95 °C for 10 min, followed by 40 cycles of sequential incubations at 95 °C for 15 s and at 60 °C for 1 min and final endpoint measurement of fluorescence. Real-Time PCR amplifications and allelic discrimination were performed using Mastercycler^®^ep realplex (Eppendorf, Hamburg, Germany).

### 2.6. Expression of the TGFBR3 Gene

Total RNA was extracted from frozen endometrial tissues using PureLink RNA Mini kit (Thermo Fisher Scientific, Waltham, MA, USA) according to dedicated protocol. The amount and quantity of isolated RNA was assessed spectrophotometrically with BioPhotometer plus (Eppendorf, Hamburg, Germany) based on A260 nm/A280 nm ratio, which was in the range 1.8–2.0. Total RNA (1 μg) was transcribed using RevertAid™ H Minus First Strand cDNA Synthesis kit (Thermo Fisher Scientific, Waltham, MA, USA) according to manufacturer’s recommendation. cDNA synthesis was performed in Thermocycler 2720 (Applied Biosystems, Foster City, CA, USA) with the following incubations: 10 min at 25 °C, 120 min at 37 °C and 5 min at 85 °C. The obtained cDNA was stored at −70 °C. Real time PCR was performed using TaqMan probes (Thermo Fisher Scientific, Waltham, MA, USA) in line with the manufacturer’s protocol on Mastercycler^®^ Epgradient S Realplex (Eppendorf, Hamburg, Germany) in the presence of TaqMan Gene Expression Master Mix Thermo Fisher Scientific, Waltham, MA, USA). *GAPDH* served as a reference gene. The catalogue numbers of probes were Hs00234259_m1 for *TGFBR*3 and Hs99999905_m1 for *GAPDH*. The relative expression level was normalized to *GAPDH* and was calculated using the following equation: 2^−ΔCt^ × 1000.

### 2.7. Statistical Analysis

The genotype frequency was tested for agreement with Hardy–Weinberg equilibrium (HWE) and assessed by chi-square goodness-of-fit test. Case-control differences in genotype and allelic distribution were analyzed using Pearson’s χ^2^ (chi-square) or Fisher’s exact tests against the homozygote of the common allele as the reference group (OR = 1.00). Dominant and recessive genetic models were also implemented in the analysis. Variants of homozygotes and heterozygotes were combined to evaluate the dominant effect. SNPs distribution and their association with the clinico-pathological parameters were evaluated by multiple logistic regression. Genotype and allelic associations with endometrial cancer risk were expressed as odds ratio (ORs) and 95% confidence interval (95% CI) in crude and multivariate model including age, BMI, parity, and age at menarche.

Linkage disequilibrium (LD) and haplotypes distribution analysis were performed using the powerful online platform SHEsis (http://analysis.bio-x.cn/myAnalysis.php) [29]. Haplotypes with frequency less than 0.03 were excluded from the analysis. Bonferroni correction was applied for multiple comparisons of SNPs haplotypes.

To assess inter-group differences of socio-demographic parameters (age, BMI, parity and menarche), as well as the *TGFBR*3 gene expression levels between respective genotypes of the analyzed SNPs, the first Shapiro–Wilk test was applied to determine the normality of obtained data. Following, the statistical significance of difference was evaluated using either Student’s t-test, for normally distributed data, or Mann–Whitney test, for non-normally distributed data. *p* < 0.05 in a two-tailed test was considered statistically significant. A statistical analysis of obtained data was conducted using GraphPad Prism version 5.00 for Windows (GraphPad Software, La Jolla, CA, USA) and PQStat version 1.6.8 (PQStat Software, Poland).

### 2.8. Bioinformatic Analysis

The functional consequences of significant SNPs were examined in RegulomeDB, which is a public database dedicated for noncoding SNP and annotates SNPs with known and putative regulatory elements in non-coding regions of human genome, such as regulatory DNA elements including regions of DNAase hypersensitivity, binding sites of transcription factors, and promoter regions that have been biochemically characterized to regulate transcription. RegulomeDB annotations are based on an integration of data from ENCODE project and other published literature, combined together by self-developed score system ranging from 1–6. A higher rank corresponds to a less functional significance [30].

## 3. Results

### 3.1. SNPs Association with EC

Eight single nucleotide polymorphisms in the *TGFBR*3 gene and their association with EC risk and invasiveness were evaluated. Three of analyzed polymorphisms, i.e., *rs883873* (g.92380302A > G), *rs2770186* (g.92378843T > C) and *rs12141128* (g.92373747A > G) are located in 5′ regulatory region, whereas the other five polymorphisms, i.e., *rs12566180* (c.−114 + 2392C > T), *rs6680463* (c.−114 + 7008C > G), *rs1805110* (p.Ser15Phe), *rs1805113* (p.Phe675=) and *rs2296621* (c.2285 − 99G > T) are located downstream start codon of the *TGFBR*3 gene.

Based on a comparison of 153 women diagnosed with endometrial cancer and 248 healthy controls, we found significant differences in the distribution of the three studied SNPs (Table 3) adjusted to the following covariates, i.e., age, BMI, menarche and parity. Significant differences between endometrial cancer patients and control women were noted in body mass index (BMI; *p* < 0.001), menarche (*p* < 0.001) and parity (*p* < 0.001). In addition, the subjects’ age showed significant differences between case and control group when analyzed in 10 years subgroups (*p* = 0.009) (Table 1). Accordingly, age, BMI, menarche and parity were selected as main covariates in further analysis. All studied SNPs were in Hardy–Weinberg equilibrium (HWE) except polymorphic site *rs1805110* (p.Ser15Phe), which was excluded from further analysis. The obtained results indicate that the polymorphisms of the highest importance for the increased endometrial cancer risk are *rs12566180* (c.−114 + 2392C > T), *rs6680463* (c.−114 + 7008C > G) and *rs2296621* (c.2285 − 99G > T). SNPs, *rs12566180* (c.-114 + 2392C > T) and *rs6680463* (c.−114 + 7008C > G) were found to be more frequent as heterozygous variants in the study group as compared to the controls with respective frequencies 55.6% vs. 44.0% (*p* = 0.0177) and 58.2% vs. 45.2% (*p* = 0.0120), increasing the risk of endometrial cancer about 2.3 times. In turn, the polymorphic site *rs2296621* (c.2285 − 99G > T) was found to be 6.4-fold more frequent in the case of study group compared to the controls (4.6% vs. 0.8%, *p* = 0.0317) as homozygous variant TT (Table 3).

Analysis of significantly altered SNPs in the cancer group in the context of clinico-pathological parameters revealed that in the case of *rs2296621* (c.2285−99G > T) the genotype GT and allele T are associated with an increased histological grade according to the WHO grading system (G3 vs. G1/2). The GT genotype was observed in 54.5% of high-graded tumors (G3) compared to 23.7% of less-graded (G1/2) (OR = 4.04; 95% CI = 1.56–10.51; *p* = 0.0026). Respectively, allele T carriers demonstrated G3 tumors more frequent than G1/2, i.e., 31.8% vs. 16.4% (OR = 2.38; 95% CI = 1.16–4.85; *p* = 0.0151) (Supplementary Table S2).

**Figure 1:**
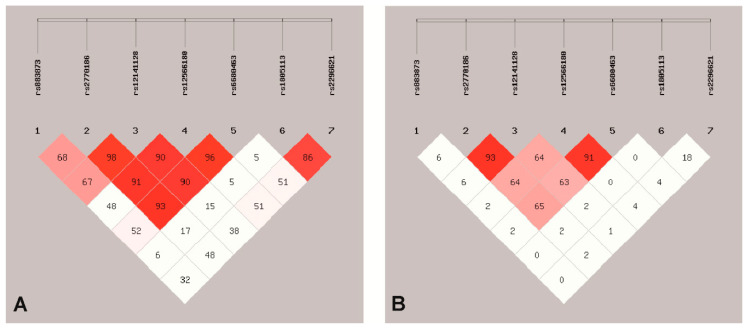
Analysis of linkage disequilibrium of *rs883873*, *rs2770186*, *rs12141128*, *rs12566180*, *rs6680463*, *rs1805113*, *rs2296621* polymorphisms in the *TGFBR*3 *gene*. (**A**). Pairwise D’ values × 100. (**B**). Pairwise r^2^ values × 100. r^2^ ≥ 0.8—high LD.

Linkage disequilibrium (LD) analysis revealed that among eight studied SNPs in the *TGFBR*3 gene, four of them were in high LD (r^2^ ≥ 0.8) (Figure 1) and were arranged in two haploblocks constructed by *rs2770186*/*rs12141128* and *rs12566180*/*rs6680463*. The frequency of haplotypes *rs12566180*/*rs6680463* C/C and T/G were significantly higher in endometrial cancer patients as compared to healthy controls: for C/C haplotype 0.038 vs. 0.008 (OR = 4.82; 95% CI = 1.54–15.07; *p* = 0.0116—Bonferroni corrected) and for T/G haplotype 0.045 vs. 0.014 (OR = 3.25; 95% CI = 1.29–8.15; *p* = 0.0328—Bonferroni corrected), respectively (Table 4).

### 3.2. Association of the TGFBR3 Gene SNPs and Betaglycan mRNA Expression—Genotype-Phenotype Analysis

**Figure 2:**
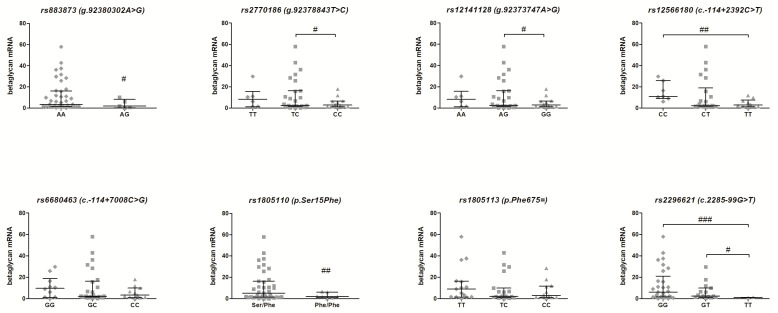
Impact of single-nucleotide polymorphisms related to the *TGFBR*3 gene on betaglycan mRNA expression in women with EC. Data are shown as scatter dot plots, *horizontal lines* represent median, whereas *whiskers* correspond to interquartile range. ^#^ *p* < 0.05, ^##^ *p* < 0.01, ^###^ *p* < 0.001.

Figure 2 shows the mRNA expression level of the *TGFBR*3 gene in 50 EC patients in relation to the genotypes of eight studied SNPs of the *TGFBR*3 gene. Six SNPs of the *TGFBR*3 gene were found to modulate its expression. The significant down-regulation of *TGFBR*3 mRNA was observed in the case of homozygous variant of *rs2770186* for genotype CC (*p* < 0.05), *rs12141128* for genotype GG (*p* < 0.05), *rs1805110* for phenotype variant Phe/Phe (*p* < 0.01) and *rs2296621* for genotype TT (*p* < 0.05) as compared to heterozygous variants. Furthermore, *rs883873* polymorphism for genotype AG (*p* < 0.05), *rs12566180* for genotype TT (*p* < 0.01) and *rs2296621* for genotype TT (*p* < 0.001) demonstrated a statistically lower *TGFBR*3 mRNA level with regard to the wild-type carriers.

## 4. Discussion

Transforming growth factors β isoforms, i.e., TGFβ1, TGFβ2 and TGFβ3, belong to a large superfamily of cytokines, which were identified due to their important role in normal development and homeostasis. TGFβ pathway controls many opposed processes, which is known as the pleiotropic effect on cell and tissue physiology. TGFβ cascade is responsible for both suppression or induction of cell proliferation and apoptosis, as well as regulates autophagy, cell dormancy and senescence. Deregulation of TGFβ signaling, both at induction step and downstream signaling contributes to developmental anomalies and diseases, in particular fibrosis and cancer, which is associated with overexpression of TGFβ isoforms [31,32]. Moreover, in cancer cells, disturbed signal mediation in TGFβ pathway triggers its role from a tumor suppressor, early in neoplastic transformation, to a cancer-promoting and metastatic agent in advanced clinical stages of the disease [33].

In cancer cells, alteration of TGFβ signaling, which plays the pleiotropic role during carcinogenesis, may be influenced by gene polymorphism. Knowledge of the potential role of polymorphism of the *TGFBR*3 gene encoding betaglycan and its relation to development of EC is elusive. Risk of endometrial cancer development is highly associated with different lifestyle and socio-demographic factors including obesity, onset of menarche, reproductive history, ethnicity and patient’s age [34,35,36,37,38,39,40]. Overweight, young age at menarche or nulliparity cause prolonged exposure to estrogens, which possess high proliferative potential, in particular to uterus lining. In the case of obesity, unopposed estrogen stimulation is the result of reduction of progesterone synthesis and higher levels of circulating estrogens. During pregnancy, the estrogen exposure is balanced by the shift toward progesterone signaling. For this reason, nulliparous women have a higher risk of developing endometrial cancer due to extended estrogen stimuli. Moreover, the increased number of births shows a protective effect on endometrial cancer occurrence [40,41,42]. Our study has demonstrated that *rs12566180* (c.−114 + 2392C > T), *rs6680463* (c.−114 + 7008C > G) and *rs2296621* (c.2285 – 99G > T) polymorphisms of the *TGFBR*3 gene are associated with increased risk of EC, both as crude or adjusted for socio-demographic risk factors, such as age, body mass index (BMI), menarche and parity. None of the patients had received EC related hormonal therapy prior to surgery; however, the patients’ records concerning hormonal replacement therapy as well as Lynch syndrome history were not available. In spite of the fact, that Lynch syndrome significantly increases the risk of EC to 25–60%, its impact on development of EC can be excluded as its occurrence ranges between 0.5% and 4.6% of all EC cases [9,10].

The *rs12566180* (c.-114 + 2392C > T) and *rs2296621* (c.2285 − 99G > T) polymorphisms significantly altered in endometrial cancer are located within intronic regions of the *TGFBR*3 gene, which may indicate their potential impact on transcription and stability of the primary transcript. Obtained results suggest that studied SNPs are involved in the observed betaglycan down-regulation in endometrial cancer; however, the only one of them, i.e., *rs2296621* (c.2285 − 99G > T), seems to be related to pronounced tumor aggressiveness. Bioinformatic analysis using RegulomeDB showed that *rs12566180* (c.-114 + 2392C > T) and *rs6680463* (c.−114 + 7008C > G) have a score of 4, whereas *rs2296621* (c.2285 − 99G > T) has a score of 2 [30]. Taking into account SNPs case-control study, haplotype analysis and genotype-phenotype findings, the observed results indicate that *rs12566180* (c.-114 + 2392C > T), *rs6680463* (c.−114 + 7008C > G) and *rs2296621* (c.2285 − 99G > T) could be regarded as potential markers for EC. However, further studies are required.

According to the literature data, polymorphism of the *TGFBR*3 gene is considered as a mechanism responsible for betaglycan down-regulation of HBV-infection related hepatocellular carcinoma and ovarian cancer [17,43,44,45]. Bae et al. [17] evaluated six SNPs, i.e., *rs1805110* (p.Ser15Phe), *rs2810904* (p.Ala72=), *rs2306888* (p.Ser173=), *rs1805113* (p.Phe675=), *rs284878* (p.Thr749=) and newly identified SNP p.Thr711=, in the *TGFBR*3 gene in a group consisting of 67 patients with hepatocellular carcinoma. In their study, *rs1805110* (p.Ser15Phe) polymorphism was found to be present at a high frequency, i.e., in about 98.5% of examined cancer samples; however, the lack of case-control comparison made it impossible to confirm the association between *rs1805110* (p.Ser15Phe) SNP occurrence and betaglycan down-regulation [17]. Similar results were presented by Xin et al. [45]. Among 16 different SNPs in genes encoding components of TGFβ pathway, i.e., TGFβ1, TGFβR1/2 and betaglycan, significantly changed frequency of *rs1805110* polymorphic site was found to be associated with incidence of HBV-related hepatocellular carcinoma (T allele, OR = 1.33; 95% CI = 1.09–1.63; *p* = 0.005) for male Chinese patients of Han ethnicity. According to the study by Kim et al. [43] *rs1805113* (Phe676Phe) in exon 13 and *rs1805117* in 3′-UTR (*p* = 0.009 and *p* = 0.008, respectively) polymorphisms were significantly associated with HBV clearance. In addition, Cox relative hazards analysis revealed that the GGTCAA haplotype of *rs2306888*, *rs1805112*, *rs1805113*, *rs284878*, *rs1805117* and *rs1804506* polymorphisms showed a significant association with the age of HCC occurrence among chronic HBV patients (relative hazard = 1.38; *p* = 0.007). In turn, the study presented by Charbonneau et al. [44] showed that in the case of mucinous invasive epithelial ovarian cancer (EOC), significantly altered distribution of polymorphisms *rs12129174* (c.384 + 1320G > A) and *rs4658265* (c.247 – 16378G > A), located in the introns of the *TGFBR*3 gene, was moderately correlated with patients’ survival (*rs12129174*—HR = 1.61; 95% CI = 1.18–2.19; *p* = 0.0038; *rs4658265*—HR = 1.56; 95% CI = 1.20–2.05; *p* = 0.0012).

Furthermore, besides above-mentioned relationship between SNPs of the *TGFBR*3 gene and neoplastic transformation, the importance of polymorphism in betaglycan encoding gene was reported for other non-cancerous diseases, i.e., premature ovarian failure (POF), testicular dysgenesis, sickle cell anemia, pulmonary emphysema and primary open angle glaucoma [46,47,48,49,50,51]. It is suggested that the *TGFBR*3 gene polymorphism may play a potential role in determining bone mineral density, as well as optic disc area parameters [52,53,54]. 

Interestingly, in our study, six out of eight analyzed SNPs of the *TGFBR*3 gene were found to have an impact on betaglycan expression in EC. Altered betaglycan expression may be responsible for impaired TGFβ signaling initiated by TGFβ isoforms and simultaneous redirection of this signal to Smad-independent pathways. *TGFBR*3 gene downregulation has been stated in the case of different cancers, and the observed decline in the expression of the *TGFBR*3 gene appears to be correlated with cancer progression, when tumor cells demonstrate an increase invasiveness and metastatic potential [17,18,19,20,21,22,23,25,26,27,28]. 

As previously described by different research groups, TGFβ signaling induced by TGFβ isoforms may be engaged in the induction of epithelial-mesenchymal transition (EMT). EMT is a biological process characterized by the reorganization of the epithelial tissue structure and is manifested by the acquisition of the mesenchymal phenotype resulting in the loss of polarity and adhesion by cells together with ability to migration and invasion. This process plays a vital role during physiological events, i.e., embryogenesis, organogenesis and morphogenesis of different tissues, wound healing, as well as inflammation. In cancer progression, EMT is responsible for the development of drug resistance and metastasis due to the increased cancer cell motility [55,56,57].

In summary, our study has demonstrated for the first time the role of the *TGFBR*3 gene polymorphism and its association with the increased risk of EC development. Moreover, we have shown that the *TGFBR*3 gene SNPs may modulate betaglycan expression at the transcriptomic level. Our findings contribute to a better understanding of the importance of gene polymorphism in the TGFβ signaling, especially at the level of signal initiation through TGFβ2 isoform mediated exclusively by betaglycan. Along with our previous findings concerning the significance of allelic loss in the *TGFBR*3 gene, where LOH was reported in 52% of examined cancer samples, SNPs may be an additional mechanism responsible for betaglycan deregulation in EC [28,58]. What is more, obtained results strongly support the view of individual variability among EC patients and suggest the necessity of developing personalized diagnostic and/or therapeutic approach in the treatment of endometrial cancer.

## Figures and Tables

**Table 1:** Socio-demographic and clinical characteristics of investigated subjects.

	Cases (*n* = 153)	Controls (*n* = 248)	*p*
**Socio-Demographic Characteristics**			
AGE (years) ^a^	62.1 ± 8.6	63.4 ± 9.0	0.1896
min	35	35	
max	85	85	
35–44 ^b^	2 (1.3)	10 (4.0)	
45–54 ^b^	25 (16.3)	36 (14.5)	
55–64 ^b^	75 (49.0)	84 (33.9)	
65–74 ^b^	38 (24.8)	78 (31.5)	
74–85 ^b^	13 (8.5)	40 (16.1)	0.009
BMI (kg/m^2^) ^a^	28.5 ± 6.6	24.9 ± 4.0	<0.001
obesity (BMI > 30 kg/m^2^) ^b^	56 (36.6)	11 (4.4)	<0.001
Menarche (years) ^a^	13.8 ± 2.0	11.9 ± 1.6	<0.001
Parity (childbirths) ^a^	1.9 ± 1.4	1.1 ± 0.9	<0.001
**Clinico-Pathological Characteristics**			
Histological Diagnosis	endometrial adenocarcinoma		
Tumor Stage ^c^			
I ^b^	89 (58.2)		
II ^b^	38 (24.8)		
III ^b^	18 (11.8)		
IV ^b^	8 (5.2)		
Histological Grade ^d^			
G1 ^b^	37 (24.2)		
G2 ^b^	94 (61.4)		
G3 ^b^	22 (14.4)		
Depth OF Myometrial Invasion			
<1/2 ^b^	82 (53.6)		
>1/2 ^b^	71 (46.4)		
Vascular Space Invasion			
not present ^b^	85 (55.6)		
present ^b^	23 (15.0)		
data not available ^b^	45 (29.4)		

BMI—body mass index, ^a^ Mean ± SD. ^b^ Number of subjects (percent total). ^c^ International Federation of Gynecology and Obstetrics staging system (FIGO). ^d^ World Health Organization grading system.

**Table 2:** Characteristics of studied polymorphisms.

rs Number	Polymorphism	Localization	Maf
*rs883873*	g.92380302A > G	5′ regulatory region	0.1394
*rs2770186*	g.92378843T > C	5′ regulatory region	0.4730
*rs12141128*	g.92373747A > G	5′ regulatory region	0.4736
*rs12566180*	c.−114 + 2392C > T	intron	0.4209
*rs6680463*	c. −114 + 7008C > G	intron	0.4687
*rs1805110*	c.44C > T (p.Ser15Phe)	exon	0.1859
*rs1805113*	c.2025T > C (p.Phe675=)	exon	0.2798
*rs2296621*	c.2285 − 99G > T	intron	0.1050

**Table 3:** Genotype distribution and allelic frequencies of investigated SNPs in the *TGFBR*3 gene for women with diagnosed endometrial cancer and healthy controls.

SNP Genotype/Allele	Cancer (*n* = 123)		Control (*n* = 248)		OR ^a^	95% CI	*p* value	OR ^b^	95% CI	*p* value	HWE ^c^
	Number	Frequency (%)	Number	Frequency (%)							
*rs883873* (g.92380302A > G)											
AA	107	69.9	200	80.6	1.00	(ref.)		1.00	(ref.)		0.3110
**AG**	45	29.4	47	19.0	**1.79**	**1.12–2.87**	**0.0149**	1.26	0.68–2.33	0.4607	
GG	1	0.7	1	0.4	1.87	0.12–30.18	1	-	-	1	
	*χ*^2^ = 6.049; *p* = **0.0486**										
AG or GG vs. AA ^d^	46		48		**1.79**	**1.12–2.86**	**0.0139**	1.28	0.70–2.37	0.4242	
AG or AA vs. GG ^e^	152		247		0.62	0.04–9.91	1	-	-	-	
**A**	259	84.6	447	90.1	**0.60**	**0.39–0.93**	**0.0203**	0.77	0.42–1.40	0.3846	
**G**	47	15.4	49	9.9	**1.66**	**1.08–2.54**	**0.0203**	1.31	0.72–2.39	0.3846	
*rs2770186* (g.92378843T > C)											
TT	22	14.4	46	18.5	1.00	(ref.)		1.00	(ref.)		0.8572
TC	89	58.2	120	48.4	1.55	0.87–2.76	0.1345	1.54	0.74–3.22	0.2469	
CC	42	27.5	82	33.1	1.07	0.57–2.01	0.8231	1.09	0.49–2.41	0.8286	
	*χ*^2^ = 3.672; *p* = 0.1595										
TC or CC vs. TT ^d^	131		202		1.36	0.78–2.36	0.2794	1.27	0.73–2.23	0.3961	
TC or TT vs. CC ^e^	111		166		1.31	0.84–2.03	0.2367	1.35	0.67–2.73	0.3930	
T	133	43.5	212	42.7	1.03	0.77–1.37	0.8415	1.02	0.70–1.50	0.9100	
C	173	56.5	284	57.3	0.97	0.73–1.29	0.8415	0.98	0.67–1.44	0.9100	
*rs12141128* (g.92373747A > G)											
AA	23	15.0	49	19.8	1.00	(ref.)		1.00	(ref.)		0.6113
AG	88	57.5	118	47.6	1.59	0.90–2.80	0.1082	1.65	0.80–3.38	0.1742	
GG	42	27.5	81	32.7	1.10	0.59–2.05	0.7518	1.14	0.52–2.47	0.7418	
	*χ*^2^*=* 3.833; *p* = 0.1471										
AG or GG vs. AA ^d^	130		199		1.39	0.81–2.39	0.2318	1.43	0.72–2.83	0.3018	
AG or AA vs. GG ^e^	111		167		1.28	0.82–2.00	0.2713	1.27	0.72–2.23	0.4023	
A	134	43.8	216	43.5	1.01	0.76–1.35	1.0000	1.00	0.68–1.46	0.9920	
G	172	56.2	280	56.5	0.99	0.74–1.32	1.0000	1.00	0.69–1.46	0.9920	
*rs12566180* (c.−114 + 2392C > T)											
CC	30	19.6	68	27.4	1.00	(ref.)		1.00	(ref.)		0.0570
**CT**	85	55.6	109	44.0	**1.77**	**1.06–2.96**	**0.0393**	**2.22**	**1.15–4.30**	**0.0177**	
TT	38	24.8	71	28.6	1.21	0.68–2.17	0.5169	1.18	0.58–2.41	0.6484	
	*χ*^2^ = 5.497; *p* = 0.0640										
CT or TT vs. CC ^d^	123		180		1.55	0.95–2.52	0.0769	1.73	0.94–3.17	0.0775	
CT or CC vs. TT ^e^	115		177		1.21	0.77–1.92	0.4062	1.42	0.80–2.51	0.2256	
C	145	47.4	245	49.4	0.92	0.69–1.23	0.5777	0.95	0.67–1.35	0.7761	
T	161	52.6	251	50.6	1.08	0.81–1.44	0.5777	1.05	0.74–1.50	0.7761	
*rs6680463* (c.−114 + 7008C > G)											
GG	29	19.0	68	27.4	1.00	(ref.)		1.00	(ref.)		0.1275
**GC**	89	58.2	112	45.2	**1.86**	**1.11–3.12**	**0.0174**	**2.34**	**1.20–4.53**	**0.0120**	
CC	35	22.9	68	27.4	1.21	0.67–2.19	0.5376	1.09	0.53–2.24	0.8243	
	*χ*^2^ = 6.758; *p* = **0.0341**										
GC or CC vs. GG ^d^	124		180		1.61	0.99–2.64	0.0544	1.74	0.95–3.20	0.0749	
GC or GG vs. CC ^e^	118		180		1.27	0.80–2.04	0.3125	1.60	0.90–2.86	0.1114	
G	147	48.0	248	50.0	0.92	0.70–1.23	0.5902	0.99	0.69–1.41	0.9470	
C	159	52.0	248	50.0	1.08	0.81–1.44	0.5902	1.01	0.71–1.45	0.9470	
*rs1805110 c.*44*C > T (p.Ser*15*Phe)*											
CC	0	0.0	0	0.0	-	-	-	-	-	-	**<0.001**
**CT**	112	73.2	212	85.5	**0.46**	**0.28–0.77**	**0.0024**	0.79	0.42–1.50	0.4771	
**TT**	41	26.8	36	14.5	**2.16**	**1.30–3.56**	**0.0024**	1.26	0.66–2.39	0.4771	
	*χ*^2^ = 9.199; *p* = **0.0100**										
CT or TT vs. CC ^d^	153		248		-	-	-	-	-	-	
**CT or CC vs. TT** ^e^	112		212		**0.46**	**0.28–0.77**	**0.0024**	0.79	0.42–1.50	0.4771	
C	112	36.6	212	42.7	0.77	0.58–1.04	0.0853	0.79	0.42–1.50	0.4771	
T	194	63.4	284	57.3	1.29	0.96–1.73	0.0853	1.26	0.66–2.39	0.4771	
*rs1805113* c.2025T > C (p.Phe675=)											
TT	56	36.6	87	35.1	1.00	(ref.)		1.00	(ref.)		0.6117
TC	68	44.4	123	49.6	0.86	0.55–1.34	0.5055	0.62	0.34–1.12	0.1115	
CC	29	19.0	38	15.3	1.19	0.66–2.14	0.5707	0.93	0.44–1.96	0.8513	
	*χ*^2^ = 1.336; *p* = 0.5127										
TC or CC vs. TT ^d^	97		161		0.94	0.62–1.42	0.7518	0.70	0.40–1.21	0.2001	
TC or TT vs. CC ^e^	124		210		0.77	0.45–1.32	0.3438	0.81	0.42–1.57	0.5405	
T	180	58.8	297	59.9	0.96	0.72–1.28	0.7642	1.10	0.77–1.58	0.6083	
C	126	41.2	199	40.1	1.04	0.78–1.40	0.7642	0.91	0.63–1.31	0.6083	
*rs2296621* c.2285 − 99G > T											
GG	103	67.3	178	71.8	1.00	(ref.)		1.00	(ref.)		0.0988
GT	43	28.1	68	27.4	1.09	0.70–1.72	0.6985	0.87	0.49–1.53	0.6226	
**TT**	7	4.6	2	0.8	**6.05**	**1.23–29.66**	**0.0178**	**6.40**	**1.18–34.84**	**0.0317**	
	*χ*^2^ = 6.272; *p* = **0.0435**										
GT or TT vs. GG ^d^	50		70		1.23	0.80–1.91	0.3428	1.04	0.60–1.80	0.8951	
**GT or GG vs. TT** ^e^	146		246		**0.17**	**0.03–0.83**	**0.0178**	**0.15**	**0.03–0.80**	**0.0267**	
G	249	81.4	424	85.5	0.74	0.51–1.09	0.1237	0.80	0.50–1.30	0.3703	
T	57	18.6	72	14.5	1.35	0.92–1.97	0.1237	1.24	0.77–2.00	0.3703	

^a^ Crude. ^b^ Adjusted for age, BMI, parity and menarche. ^c^ Hardy–Weinberg equilibrium test for controls. ^d^ Testing dominant genetic model. ^e^ Testing recessive genetic model; *p* < 0.05 along with corresponding ORs are in bold.

**Table 4:** Distribution of combined haplotype of *rs12566180*, *rs6680463* and *rs2296621* polymorphisms and risk of the endometrial cancer. (Bold indicates statistically significant results.).

Combined Haplotype	Cancer		Control		OR	95% CI	*p* Value (Bonferroni Corrected)
	Number	Frequency (%)	Number	Frequency (%)			
*rs2770186*/*rs12141128*							
C/A	1	0.003	6.02	0.012	-	-	-
C/G	172	0.562	277.98	0.560	0.98	0.73–1.30	1.0000
T/A	133	0.435	209.98	0.423	1.02	0.77–1.37	1.0000
T/G	0	0.000	2.02	0.004	-	-	-
*rs12566180*/*rs6680463*							
**C/C**	11.69	0.038	4.05	0.008	**4.82**	**1.54–15.07**	**0.0116**
C/G	133.31	0.436	240.95	0.486	0.82	0.61–1.09	0.6676
T/C	147.31	0.481	243.95	0.492	0.96	0.72–1.28	1.0000
**T/G**	13.69	0.045	7.05	0.014	**3.25**	**1.29–8.15**	**0.0328**

## Supplementary Materials

Table S1: Characteristics and sequences of TaqMan probes used for genotyping of *TGFBR*3 gene; Table S2: The association between significantly altered SNPs, i.e., *rs12566180*, *rs6680463* and *rs2296621* polymorphisms and clinico-pathological parameters of studied cancer samples.

## References

[1] Ferlay J., Colombet M., Soerjomataram I., Mathers C., Parkin D.M., Piñeros M., Znaor A., Bray F. (2019). Estimating the global cancer incidence and mortality in 2018: GLOBOCAN sources and methods. Int. J. Cancer.

[2] Didkowska J., Wojciechowska U., Czaderny K., Olasek P., Ciuba A. (2019). Cancer in Poland in 2017.

[3] Bokhman J.V. (1983). Two pathogenetic types of endometrial carcinoma. Gynecol. Oncol..

[4] Webb P.M. (2015). Environmental (nongenetic) factors in gynecological cancers: Update and future perspectives. Future Oncol..

[5] Murali R., Soslow R.A., Weigelt B. (2014). Classification of endometrial carcinoma: More than two types. Lancet Oncol..

[6] Yen T.T., Wang T.L., Fader A.N., Shih I.M., Gaillard S. (2020). Molecular Classification and Emerging Targeted Therapy in Endometrial Cancer. Int. J. Gynecol. Pathol..

[7] Lee Y.C., Lheureux S., Oza A.M. (2017). Treatment strategies for endometrial cancer: Current practice and perspective. Curr. Opin. Obstet. Gynecol..

[8] Getz G., Gabriel S.B., Cibulskis K., Lander E., Sivachenko A., Sougnez C., Lawrence M., Kandoth C., Dooling D., Fulton R. (2013). Integrated genomic characterization of endometrial carcinoma. Nature.

[9] Bell D.W., Ellenson L.H. (2019). Molecular Genetics of Endometrial Carcinoma. Annu. Rev. Pathol. Mech. Dis..

[10] Rossi L., Le Frere-Belda M.A., Laurent-Puig P., Buecher B., De Pauw A., Stoppa-Lyonnet D., Canlorbe G., Caron O., Borghese B., Colas C. (2017). Clinicopathologic characteristics of endometrial cancer in lynch syndrome A French multicenter study. Int. J. Gynecol. Cancer.

[11] Parekh T.V., Gama P., Wen X., Demopoulos R., Munger J.S., Carcangiu M.L., Reiss M., Gold L.I. (2002). Transforming growth factor β signaling is disabled early in human endometrial carcinogenesis concomitant with loss of growth inhibition. Cancer Res..

[12] Piestrzeniewicz-Ulanska D., Brys M., Semczuk A., Jakowicki J.A., Krajewska W.M. (2002). Expression of TGF-β type I and II receptors in normal and cancerous human endometrium. Cancer Lett..

[13] Piestrzeniewicz-Ulanska D., Brys M., Semczuk A., Rechberger T., Jakowicki J.A., Krajewska W.M. (2004). TGF-β signaling is disrupted in endometrioid-type endometrial carcinomas. Gynecol. Oncol..

[14] Drabsch Y., Ten Dijke P. (2012). TGF-β signalling and its role in cancer progression and metastasis. Cancer Metastasis Rev..

[15] Gordon K.J., Blobe G.C. (2008). Role of transforming growth factor-β superfamily signaling pathways in human disease. Biochim. Biophys. Acta Mol. Basis Dis..

[16] Cheifetz S., Andres J.L., Massagué J. (1988). The transforming growth factor-beta receptor type III is a membrane proteoglycan. Domain structure of the receptor. J. Biol. Chem..

[17] Bae H.J., Eun J.W., Noh J.H., Kim J.K., Jung K.H., Xie H.J., Park W.S., Lee J.Y., Nam S.W. (2009). Down-regulation of transforming growth factor β receptor type III in hepatocellular carcinoma is not directly associated with genetic alterations or loss of heterozygosity. Oncol. Rep..

[18] Bilandzic M., Chu S., Farnworth P.G., Harrison C., Nicholls P., Wang Y., Escalona R.M., Fuller P.J., Findlay J.K., Stenvers K.L. (2009). Loss of betaglycan contributes to the malignant properties of human granulosa tumor cells. Mol. Endocrinol..

[19] Copland J.A., Luxon B.A., Ajani L., Maity T., Campagnaro E., Guo H., LeGrand S.N., Tamboli P., Wood C.G. (2003). Genomic profiling identifies alterations in TGFβ signaling through loss of TGFβ receptor expression in human renal cell carcinogenesis and progression. Oncogene.

[20] Dong M., How T., Kirkbride K.C., Gordon K.J., Lee J.D., Hempel N., Kelly P., Moeller B.J., Marks J.R., Blobe G.C. (2007). The type III TGF-β receptor suppresses breast cancer progression. J. Clin. Investig..

[21] Finger E.C., Turley R.S., Dong M., How T., Fields T.A., Blobe G.C. (2008). TβRIII suppresses non-small cell lung cancer invasiveness and tumorigenicity. Carcinogenesis.

[22] Gordon K.J., Dong M., Chislock E.M., Fields T.A., Blobe G.C. (2008). Loss of type III transforming growth factor beta receptor expression increases motility and invasiveness associated with epithelial to mesenchymal transition during pancreatic cancer progression. Carcinogenesis.

[23] Hempel N., How T., Dong M., Murphy S.K., Fields T.A., Blobe G.C. (2007). Loss of betaglycan expression in ovarian cancer: Role in motility and invasion. Cancer Res..

[24] Iolascon A., Giordani L., Borriello A., Carbone R., Izzo A., Tonini G.P., Gambini C., Della Ragione F. (2000). Reduced expression of transforming growth factor-beta receptor type III in high stage neuroblastomas. Br. J. Cancer.

[25] Liu X.L., Xiao K., Xue B., Yang D., Lei Z., Shan Y., Zhang H.T. (2013). Dual role of TGFBR3 in bladder cancer. Oncol. Rep..

[26] Sharifi N., Hurt E.M., Kawasaki B.T., Farrar W.L. (2007). TGFBR3 loss and consequences in prostate cancer. Prostate.

[27] Turley R.S., Finger E.C., Hempel N., How T., Fields T.A., Blobe G.C. (2007). The type III transforming growth factor-beta receptor as a novel tumor suppressor gene in prostate cancer. Cancer Res..

[28] Zakrzewski P.K., Mokrosinski J., Cygankiewicz A.I., Semczuk A., Rechberger T., Skomra D., Krajewska W.M. (2011). Dysregulation of betaglycan expression in primary human endometrial carcinomas. Cancer Investig..

[29] Shi Y.Y., He L. (2005). SHEsis, a powerful software platform for analyses of linkage disequilibrium, haplotype construction, and genetic association at polymorphism loci. Cell Res..

[30] Boyle A.P., Hong E.L., Hariharan M., Cheng Y., Schaub M.A., Kasowski M., Karczewski K.J., Park J., Hitz B.C., Weng S. (2012). Annotation of functional variation in personal genomes using RegulomeDB. Genome Res..

[31] Akhurst R.J. (2017). Targeting TGF-β Signaling for Therapeutic Gain. Cold Spring Harb. Perspect. Biol..

[32] Zhang Y., Alexander P.B., Wang X.F. (2017). TGF-β family signaling in the control of cell proliferation and survival. Cold Spring Harb. Perspect. Biol..

[33] Xie F., Ling L., Van Dam H., Zhou F., Zhang L. (2018). TGF-β signaling in cancer metastasis. Acta Biochim. Biophys. Sin..

[34] Jenabi E., Poorolajal J. (2015). The effect of body mass index on endometrial cancer: A meta-analysis. Public Health.

[35] Gao Y., Dai X., Chen L., Lee A.C., Tong M., Wise M., Chen Q. (2016). Body Mass Index Is Positively Associated with Endometrial Cancer in Chinese Women, Especially Prior to Menopause. J. Cancer.

[36] Althubiti M. (2019). Mutation frequencies in endometrial cancer patients of different ethnicities and tumor grades: An analytical study. Saudi J. Med. Med. Sci..

[37] Park S.L., Goodman M.T., Zhang Z.F., Kolonel L.N., Henderson B.E., Setiawan V.W. (2010). Body size, adult BMI gain and endometrial cancer risk: The multiethnic cohort. Int. J. Cancer.

[38] Garg K., Soslow R.A. (2014). Endometrial carcinoma in women aged 40 years and younger. Arch. Pathol. Lab. Med..

[39] O’Mara T.A., Glubb D.M., Kho P.F., Thompson D.J., Spurdle A.B. (2019). Genome-Wide Association Studies of Endometrial Cancer: Latest Developments and Future Directions. Cancer Epidemiol. Biomark. Prev..

[40] Chen Q., Tong M., Guo F., Lau S., Zhao M. (2015). Parity correlates with the timing of developing endometrial cancer, but not subtype of endometrial cancer. J. Cancer.

[41] Karageorgi S., Hankinson S.E., Kraft P., De Vivo I. (2010). Reproductive factors and postmenopausal hormone use in relation to endometrial cancer risk in the Nurses’ Health Study cohort 1976–2004. Int. J. Cancer.

[42] Dossus L., Allen N., Kaaks R., Bakken K., Lund E., Tjonneland A., Olsen A., Overvad K., Clavel-Chapelon F., Fournier A. (2010). Reproductive risk factors and endometrial cancer: The European prospective investigation into cancer and nutrition. Int. J. Cancer.

[43] Kim J.-H., Jong S., Park B.-L., Sub H., Yu S.J., Cheong H.S., Pasaje C.F.A., Bae J.S., Lee H.-S., Shin H.D. (2011). TGFBR3 Polymorphisms and Its Haplotypes Associated with Chronic Hepatitis B Virus Infection and Age of Hepatocellular Carcinoma. Dig. Dis..

[44] Charbonneau B., Moysich K.B., Kalli K.R., Oberg A.L., Vierkant R.A., Fogarty Z.C., Block M.S., Maurer M.J., Goergen K.M., Fridley B.L. (2014). Large-scale evaluation of common variation in regulatory T cell-related genes and ovarian cancer outcome. Cancer Immunol. Res..

[45] Xin Z., Zhang W., Xu A., Zhang L., Yan T., Li Z., Wu X., Zhu X., Ma J., Li K. (2012). Polymorphisms in the potential functional regions of the TGF-β 1 and TGF-β receptor genes and disease susceptibility in HBV-related hepatocellular carcinoma patients. Mol. Carcinog..

[46] Chand A.L., Robertson D.M., Shelling A.N., Harrison C.A. (2007). Mutational analysis of betaglycan/TGF-βRIII in premature ovarian failure. Fertil. Steril..

[47] Dalgaard M.D., Weinhold N., Edsgärd D., Silver J.D., Pers T.H., Nielsen J.E., Jørgensen N., Juul A., Gerds T.A., Giwercman A. (2012). A genome-wide association study of men with symptoms of testicular dysgenesis syndrome and its network biology interpretation. J. Med. Genet..

[48] Elliott L., Ashley-Koch A.E., De Castro L., Jonassaint J., Price J., Ataga K.I., Levesque M.C., Brice Weinberg J., Eckman J.R., Orringer E.P. (2007). Genetic polymorphisms associated with priapism in sickle cell disease. Br. J. Haematol..

[49] Flanagan J.M., Frohlich D.M., Howard T.A., Schultz W.H., Driscoll C., Nagasubramanian R., Mortier N.A., Kimble A.C., Aygun B., Adams R.J. (2011). Genetic predictors for stroke in children with sickle cell anemia. Blood.

[50] Hersh C.P., Hansel N.N., Barnes K.C., Lomas D.A., Pillai S.G., Coxson H.O., Mathias R.A., Rafaels N.M., Wise R.A., Connett J.E. (2009). Transforming growth factor-β receptor-3 is associated with pulmonary emphysema. Am. J. Respir. Cell Mol. Biol..

[51] Li Z., Allingham R.R., Nakano M., Jia L., Chen Y., Ikeda Y., Mani B., Chen L.J., Kee C., Garway-Heath D.F. (2015). A common variant near TGFBR3 is associated with primary open angle glaucoma. Hum. Mol. Genet..

[52] Duncan E.L., Danoy P., Kemp J.P., Leo P.J., McCloskey E., Nicholson G.C., Eastell R., Prince R.L., Eisman J.A., Jones G. (2011). Genome-wide association study using extreme truncate selection identifies novel genes affecting bone mineral density and fracture risk. PLoS Genet..

[53] Khor C.C., Ramdas W.D., Vithana E.N., Cornes B.K., Sim X., Tay W.T., Saw S.M., Lavanya Y.Z., Wu R., Wang J.J. (2011). Genome-wide association studies in Asians confirm the involvement of ATOH7 and TGFBR3, and further identify CARD10 as a novel locus influencing optic discarea. Hum. Mol. Genet..

[54] Xiong D.H., Liu X.G., Guo Y.F., Tan L.J., Wang L., Sha B.Y., Tang Z.H., Pan F., Yang T.L., Chen X.D. (2009). Genome-wide Association and Follow-Up Replication Studies Identified ADAMTS18 and TGFBR3 as Bone Mass Candidate Genes in Different Ethnic Groups. Am. J. Hum. Genet..

[55] Hanahan D., Weinberg R.A. (2011). Hallmarks of cancer: The next generation. Cell.

[56] Makker A., Goel M.M. (2016). Tumor progression, metastasis, and modulators of epithelial-mesenchymal transition in endometrioid endometrial carcinoma: An update. Endocr. Relat. Cancer.

[57] Zhang J., Tian X.-J., Xing J. (2016). Signal Transduction Pathways of EMT Induced by TGF-β, SHH, and WNT and Their Crosstalks. J. Clin. Med..

[58] Zakrzewski P.K., Nowacka-Zawisza M., Semczuk A., Rechberger T., Gałczyński K., Krajewska W.M. (2016). Significance of TGFBR3 allelic loss in the deregulation of TGF signaling in primary human endometrial carcinomas. Oncol. Rep..

